# Impact of EEG Frequency Bands and Data Separation on the Performance of Person Verification Employing Neural Networks

**DOI:** 10.3390/s22155529

**Published:** 2022-07-25

**Authors:** Renata Plucińska, Konrad Jędrzejewski, Marek Waligóra, Urszula Malinowska, Jacek Rogala

**Affiliations:** 1 Institute of Electronic Systems, Faculty of Electronics and Information Technology, Warsaw University of Technology, 00-665 Warsaw, Poland; renata.plucinska.dokt@pw.edu.pl; 2 Laboratory of Neuroinformatics, Nencki Institute of Experimental Biology, 02-093 Warsaw, Poland; waligora.marek@gmail.com (M.W.); u.malinowska@nencki.edu.pl (U.M.); 3 Institute of Physiology and Pathology of Hearing, Bioimaging Research Center, World Hearing Center, Kajetany, 05-830 Nadarzyn, Poland; rogala.jacek@gmail.com

**Keywords:** EEG, electroencephalography, biometry, verification, neural network

## Abstract

The paper is devoted to the study of EEG-based people verification. Analyzed solutions employed shallow artificial neural networks using spectral EEG features as input representation. We investigated the impact of the features derived from different frequency bands and their combination on verification results. Moreover, we studied the influence of a number of hidden neurons in a neural network. The datasets used in the analysis consisted of signals recorded during resting state from 29 healthy adult participants performed on different days, 20 EEG sessions for each of the participants. We presented two different scenarios of training and testing processes. In the first scenario, we used different parts of each recording session to create the training and testing datasets, and in the second one, training and testing datasets originated from different recording sessions. Among single frequency bands, the best outcomes were obtained for the beta frequency band (mean accuracy of 91 and 89% for the first and second scenarios, respectively). Adding the spectral features from more frequency bands to the beta band features improved results (95.7 and 93.1%). The findings showed that there is not enough evidence that the results are different between networks using different numbers of hidden neurons. Additionally, we included results for the attack of 23 external impostors whose recordings were not used earlier in training or testing the neural network in both scenarios. Another significant finding of our study shows worse sensitivity results in the second scenario. This outcome indicates that most of the studies presenting verification or identification results based on the first scenario (dominating in the current literature) are overestimated when it comes to practical applications.

## 1. Introduction

Electroencephalography (EEG) is a widely used method for recording bioelectrical brain activity. Interpretation of EEG signals is not a simple task, but it became a helpful tool in neuroscience research because of its high temporal resolution. The primary sources of the EEG signals are nerve cells (neurons) located in the cerebral cortex. Neurons receive and transmit signals in short electrical impulses (spikes), producing waves of electrical currents registered by sensors located on the scalp. The results of recorded EEG signals are represented by the changes in the voltage collected by electrodes. Advanced methods for processing such signals are one of the fastest-growing fields of science and technology in biomedical engineering.

EEG is widely used in medicine and neuroscience research, brain–computer interfaces (BCIs) [1], and EEG-neurofeedback [2]. However, those signals are strongly contaminated by environmental and physiological artifacts [3]. Difficulties in interpreting the EEG signals also arise from intra- and inter-individual variability, non-stationarity of the signals [4], and volume conduction [5]. The rapid development of machine learning (ML) methods and artificial neural networks (ANNs) offers possibilities to overcome these obstacles.

Artificial neural network models can be successfully used for EEG classification of any living person (cross-participant models). ML and ANN methods have been effectively used for the classification of epilepsy [6] and to identify patterns of mental disorders, such as attention deficit hyperactivity disorder (ADHD) [7]. In recent years, they have also been used in biomedical engineering, including emotion recognition [8].

The use of the EEG and ML methods has also been considered to be employed in biometrics for verification (confirmation of claimed identity) and identification (searching for identity in a biometric enrolment database) [9]. The EEG signals, compared to some other biometrics (e.g., fingerprint), can only be provided by a living person, so they have liveness detection [10] and cannot be captured at a distance [11]. Moreover, EEG is vulnerable to a wide range of stimuli, which can help detect abnormal states in the signals of a person being recognized [12]. The use of EEG biometrics was widely described in [12,13].

There are many methods for EEG-based biometry recognition [13,14,15] based on analysis of evoked potentials or analysis of task performance during EEG recordings. In [14,15,16], EEG-based biometric recognition systems were classified based on resting states with eyes open (REO) or eyes closed (REC), event-related potentials (ERPs), and intentional cognitive activities. Recently, much attention has been paid to systems based on ERPs, where the reaction to external stimuli is analyzed (for example, visual stimuli, as in [16,17]) and mental tasks, such as in [18]. However, in our study, we decided to analyze REO signals, where a subject was instructed to sit still with eyes open without performing any intentional mental activity. The rationale behind our choice is threefold: first, resting-state EEG is an easy paradigm in a practical application (with no need for additional special software and hardware during acquisition), which is of importance in biometrics. Second, it is the most popular protocol for acquiring EEG signals (for more information, see [13]). Third, features of resting-state EEG are highly heritable [19,20], which indicates that resting-state EEG consists of permanent individual features, which can be used in biometrics. The resting-state paradigm has high research importance, starting from the seminal discovery of fMRI resting-state connectivity by [21], and resting-state investigations both in fMRI and EEG/MEG are the core studies of the human connectome delivering hundreds of important observations and discoveries.

In the late 1990s, the authors of [22] successfully identified individuals based on resting state recordings with eyes closed, which became the basis for further EEG-based biometric research. In these studies, the authors recorded 45 sessions from four subjects and one session from 75 subjects using one EEG channel. Despite using only spectral features of the alpha frequency band, the authors obtained promising results (in Test Case #1, the sensitivity ranged from 80 to 100% and the specificity from 80 to 90%, for different subjects and the alpha frequency subband).

Over a decade later, in 2010, the authors of [23] managed to achieve for four channels a correct recognition rate (CRR) of 78–81%, depending on whether the eyes were open or closed, and, for selected channels, 54–71% for eyes open and 64–68% for eyes closed. The authors used the wavelet packet decomposition and a neural network during their studies. In the study conducted, 90% of the data were used for training and the remaining 10% for testing. Although this work is about identification, the studies were performed on 10 participants with five separate sessions conducted over two weeks.

In [15], among the techniques of extraction of EEG features, the authors distinguished methods based on: power spectral density (PSD), autoregressive models (ARs), wavelet transform (WT), Hilbert–Huang Transform (HHT), and others. They also described classification methods based on the k-nearest neighbor (k-NN) algorithms, linear discriminate analysis (LDA), artificial neural networks (ANNs), and kernel methods, e.g. support vector machine (SVM). In [12,13], the authors described analysis based on time, frequency, time–frequency, spatial domains, and non-linear dynamics methods. They also presented methods based on AR parameter models, PSD, WT, common space patterns, and phase synchronization. In classification, they distinguished methods based on shallow classification such as LDA, SVM, low-rank sparse decomposition, Bayesian networks, ANNs, and more. Methods based on deep learning were also characterized. It should be noted that although there are many methods of EEG signal processing for biometric applications, in this paper, we focused on the usage of EEG spectral characteristics.

The practical application of biometrics assumes multiple verifications or identifications of the same person across many days. However, most of the studies used data from a single EEG recording session [10,17,18,24,25], from recording sessions collected on the same day [26], or for a small number of people with multiple recording sessions [22]. Some works provided a limited number of recording sessions [23,27,28,29,30]. It is suggested that most studies focus on analyzing inter-individual differences rather than their stability over time [11]. These procedures cannot reflect the possible application of EEG biometrics in practice. While excellent results are obtained when discriminating between individuals, the results deteriorate as the number of subjects increases [12,13]. In [31], it was shown that by adding more subjects, the accuracy of the system could be drastically reduced. Many articles mentioned the need to research with a more extensive database (e.g., [11,13,14,32,33]). Some longitudinal analyses on the five different sessions with 45 healthy subjects were performed in [30].

In person verification or identification, it is necessary to ensure the constancy of the recognized parameters over time. For this reason, we analyzed how the method of data splitting into the training and testing datasets can influence the efficiency of EEG-based biometrics verification. We checked whether the research on the performance of different EEG-based verification methods should be carried out using separate (especially over time) recordings or whether we can use recordings from each of the sessions for training and testing. As mentioned above, many studies found in the literature were conducted in a paradigm focused more on differences between the participants than the solution’s stability and tested the classifier’s performance with the training data from the same EEG recordings. We decided to verify the hypothesis that obtaining high biometric recognition metrics is directly due to the use of the same dataset in the analyses and could indicate a problematic data leakage effect known in the field of machine learning [34].

To demonstrate the effects of these two different approaches to dataset handling on the accuracy, sensitivity, specificity, and precision of a multi-participant verification model, we compared the performance of two verification paradigms (scenarios). In the first, we used the different parts of each recorded EEG session in the training and testing process. In the second, the training and testing process datasets were separated by EEG sessions. Our research examined the impact of the used scenario on the number of hidden neurons and the set of EEG frequency bands and their combinations that will provide the best results. An essential element of our research was organizing a sufficient number of EEG recordings from many days with numerous participants.

## 2. Materials and Methods

### 2.1. Data Collection

The studies were conducted using EEG recordings obtained from 29 participants (14 females and 15 males, mean age 28.17 (23–44)). Each participant attended 20 EEG sessions scattered over an average of approximately 70 (43–129) days, resulting in 580 separate examinations. We also included individual recordings from an additional 23 people to simulate external impostor attacks. The sampling frequency of the EEG recordings was 500 Hz. Electrodes were positioned following the 10–20 international electrode placement system [35], and the impedance was kept below 10 kΩ. The ExG-32 headbox manufactured by ELMIKO BIOSIGNALS was used to acquire signals. The reference A2 electrode was placed on the right ear. All participants were right-handed and had normal or corrected to normal vision.

Our verification methods were based on neural networks trained to classify specific spectral features in the canonical EEG frequency bands: delta δ (1–4 Hz), theta θ (4–8 Hz), alpha α (8–12 Hz), beta β (12–30 Hz), and gamma γ (30–45 Hz). Further in the text, the Greek letters will be used for each EEG frequency band. The upper border of the γ frequency band was set to 45 Hz to avoid signal contamination by the 50 Hz power line interferences.

### 2.2. Feature Extraction

Three minutes from each EEG examination were selected for analysis. For preprocessing, we applied the common average reference (CAR), i.e., from the signal at each electrode, the mean signal of all electrodes was subtracted, as described in [13]. Following the results in [10,24,36], every examination was divided into 7.5 s segments. Next, Welch’s power spectral density was estimated for each segment, with a 1s sliding Hamming window and an overlap of 0.5 s. We did not perform other preprocessing methods or artifact removal to imitate the conditions of the practical application of the considered verification method.

After evaluating the different sets of spectral features, their inter-correlations, and the minimal number to obtain satisfactory results, the following set of features was selected for all frequency bands and all channels:

- the normalized peak frequency (the peak frequency divided by the sampling frequency);
- the peak power in the frequency band divided by the mean power in this frequency band;
- the total power in the frequency band divided by the total signal power.

**Figure 1:**
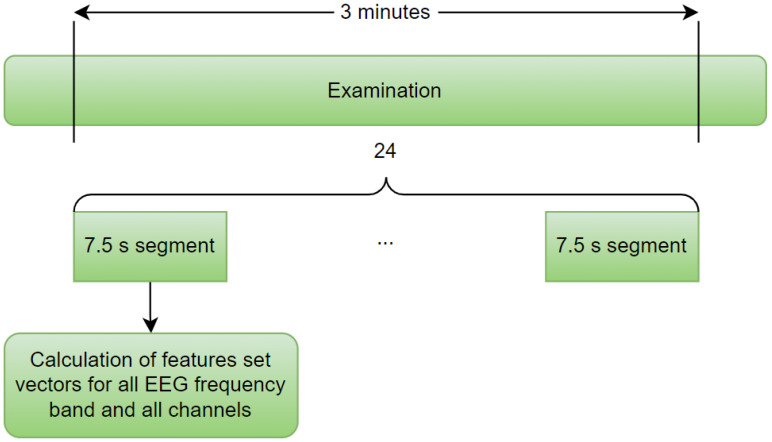
Data preparation path.

There were 57 input features per single EEG band and 285 features in the maximum configuration. The data preparation method is presented in Figure 1.

### 2.3. Neural Networks

Taking into account the set of considered features and the potential applicability in practical EEG-based verification systems, we decided to use feedforward neural networks [37] with one hidden layer and two output neurons. A sigmoidal hyperbolic tangent transfer function was used in the hidden layer and a linear one in the output layer. Each network was trained using the Levenberg–Marquardt backpropagation algorithm [38]. As we investigated the person verification (confirmation of claimed identity), a separate neural network was created and trained for each participant to recognize them. The same recordings from 29 subjects were used in both scenarios. We performed the two-class classification, where genuine and impostor data were used for training. Each time one of these subjects was verified (using their own network as a biometric reference), the impostor data for the network training and testing were randomly drawn from the data of the other 28 subjects.

**Figure 2:**
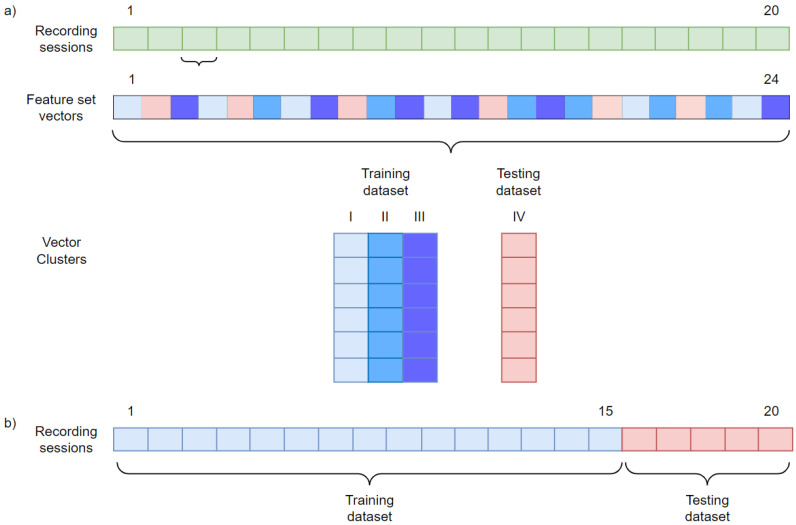
The division of data into training and testing sets for a person being verified in the first (**a**) and second (**b**) scenario. An exemplary set for testing—red, blue—for training.

In both analyzed scenarios (first—different parts of each recorded EEG session used for training and testing, second—the training and testing datasets from different EEG sessions), we used all 20 recording sessions. For each session, three-minute EEG signals were split into 24 segments with a length of 7.5 s. For each segment, the feature set vectors were calculated (Figure 1). The division into training and testing datasets in the first scenario is presented in Figure 2a. The training and testing dataset features vectors were selected randomly from different parts of the same recordings. For the cross-validation, the order of these vectors was randomized and then divided into four equal vector clusters, which resulted in six vectors in each vector cluster. One was used to test each verified person, while the others were for training. The feature set vectors for impostors were randomly selected, and their number was established to balance the number of positive and negative cases in the training process.

The dataset separation in the second scenario is presented in Figure 2b. Feature set vectors for the training and testing datasets were drawn from recording sessions performed on different days. For each person, the vectors obtained from the first 15 recordings were used for training and from the last 5 for testing. The feature set vectors from the impostor’s recording were randomly selected four times to perform the cross-validation. The number of selected feature sets for impostors was the same as for the verified people.

### 2.4. Results Evaluation

We evaluated the results of the verification procedures using common statistical classification measures:

(1) $$\mathrm{accuracy}:\;\mathrm{ACC}=\frac{\mathrm{TP}+\mathrm{TN}}{\mathrm{TP}+\mathrm{FN}+\mathrm{FP}+\mathrm{TN}},$$

(2) $$\mathrm{sensitivity}:\;\mathrm{SEN}=\frac{\mathrm{TP}}{\mathrm{TP}+\mathrm{FN}},$$

(3) $$\mathrm{specificity}:\;\mathrm{SPEC}=\frac{\mathrm{TN}}{\mathrm{FP}+\mathrm{TN}},$$

(4) $$\mathrm{precision}:\;\mathrm{PREC}=\frac{\mathrm{TP}}{\mathrm{TP}+\mathrm{FP}},$$

where:

- true positive (TP) is the number of segments (feature set vectors) adequately recognized as genuine (a verified person);
- true negative (TN) is the number of segments correctly classified as an impostor (a person who pretends to be the verified person);
- false positive (FP) is the number of segments incorrectly classified as genuine;
- false negative (FN) is the number of segments incorrectly classified as an impostor.

In this study, accuracy can be described as the factor of correctly classified segments, sensitivity as the ability of a system to detect a genuine person, specificity is the ability to detect an impostor, and precision is the factor of correctly classified genuine people from all recognized as a genuine claimant.

## 3. Results

### 3.1. Influence of the Number of Hidden Neurons on the Verification Performance for Both Scenarios

We started with assessing the number of neurons in the hidden layer on the authentication accuracy for both scenarios. We investigated the verification performance of individual frequencies and, based on these results, we selected combinations of the frequency bands for further investigations.

For both scenarios, the number of hidden neurons varied from 1 to 10. For each number of hidden neurons and vector cluster separation (see Figure 2 for details), 10 networks were created. The one with the highest accuracy in the training dataset was selected for each person. The average accuracy (averaged over all participants) and standard deviation for each EEG frequency band set for the first scenario (feature set vectors for training and testing datasets extracted from each recording session) are presented in Table 1. The results for the second scenario (feature vectors for both datasets extracted from different sessions) are shown in Table 2. Having analyzed further results for single frequency bands, we decided to combine the β band with the adjacent ones.

We performed a two-way analysis of variance (ANOVA) to compare for each scenario the averaged accuracy values for different frequency bands and different numbers of hidden neurons. In both scenarios, the ANOVA results show that there is not enough evidence that the results are different for the analyzed numbers of hidden neurons (ANOVA F-test (9, 2800) = 0.23, *p* > 0.99 for the first scenario and F (9, 2800) = 0.07, *p* > 0.99 for the second) and for a strong effect of the considered EEG frequency bands on the achieved accuracy (first scenario: F (9, 2800) = 286.41, *p* = 0, second scenario: F (9, 2800) = 138.19, *p* = 0). Moreover, we did not find enough evidence for significant interactions between factors (first scenario: F (9, 2800) = 0.19, *p* = 1, second scenario: F (9, 2800) = 0.08, *p* = 1).

**Figure 3:**
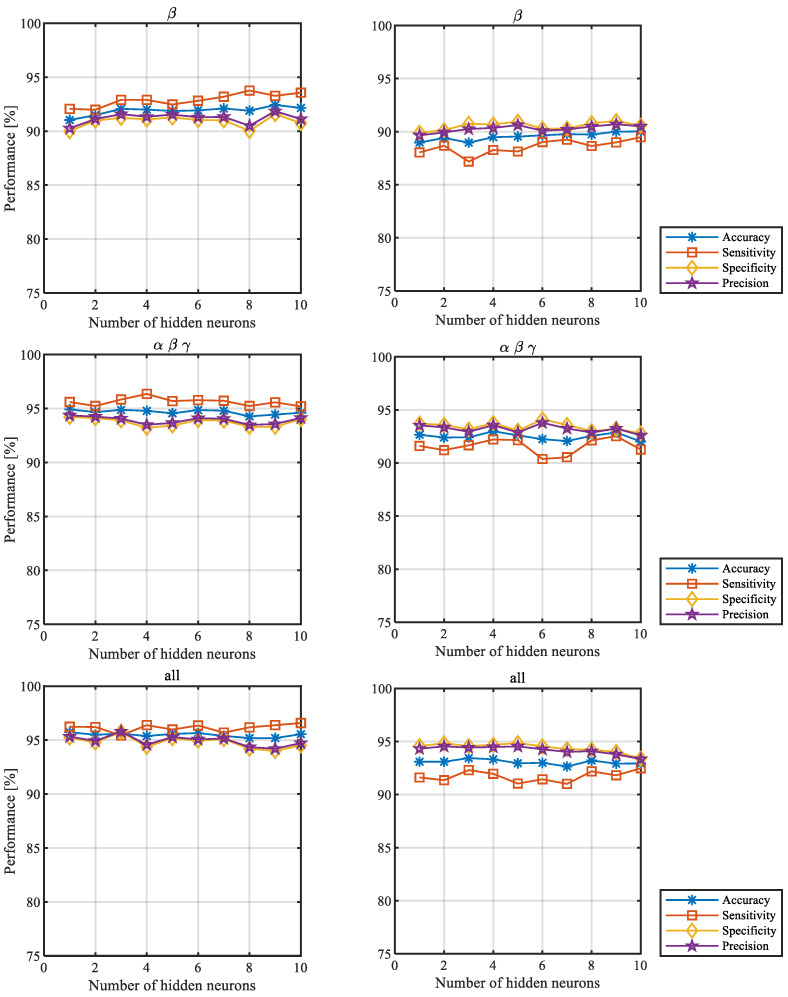
Network classification measures for selected frequency bands, where the training and testing feature set vectors were separated by different parts of the same sessions (**left**, the first scenario) and different sessions (**right**, the second scenario).

**Figure 4:**
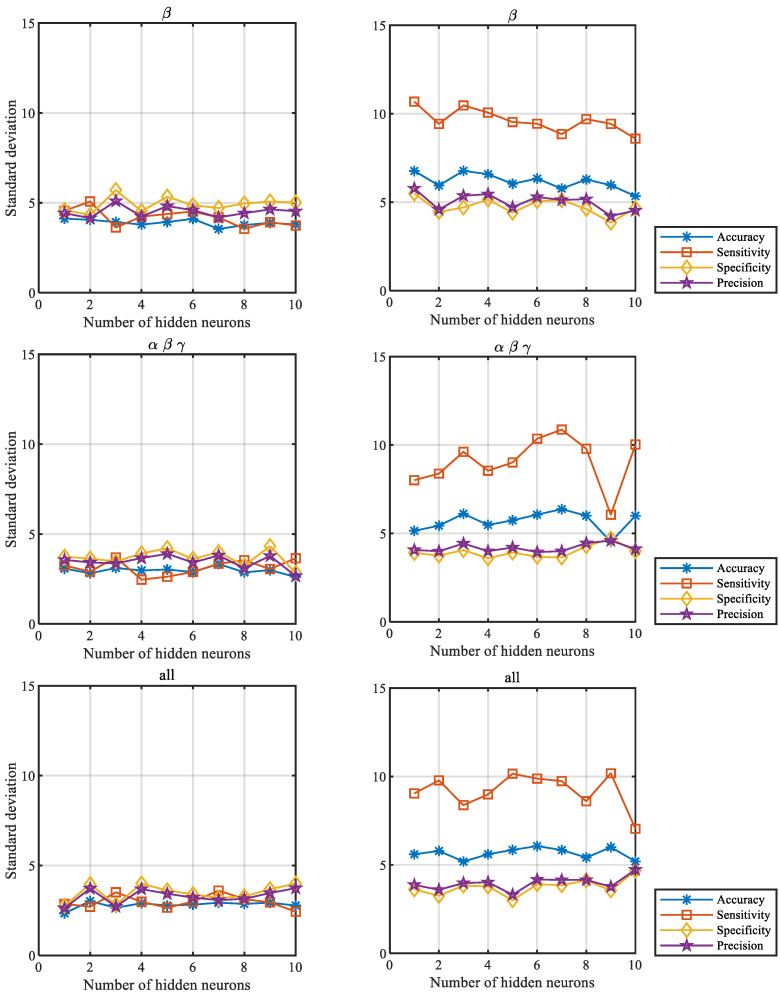
Standard deviations of network classification measures for selected frequency bands, where the training and testing feature set vectors were separated by different parts of the same sessions (**left**, the first scenario) and by different sessions (**right**, the second scenario).

To further illustrate the stability of the results depending on the number of hidden neurons, we determined the outcomes obtained for all network classification measures: accuracy, sensitivity, specificity, and precision, described in Section 2.4. The values of the measures for different numbers of hidden neurons obtained for the first scenario (left column) and the second one (right column) are presented in Figure 3. Their standard deviations are presented in Figure 4. The results were averaged over all participants and correspond to the number of hidden neurons ranging from 1 to 10.

The figures below show the averaged values for the β frequency band, a combination of α, β, and γ frequency bands, and all analyzed frequency bands. For the results of the remaining frequency bands and their combinations, see Appendix A.

Since there is not enough evidence that the results for different numbers of hidden neurons are different, we further analyzed the results for one hidden neuron. This choice is justified by practical issues related to optimizing the computation time.

### 3.2. Performance of the Models Trained with One Hidden Neuron

We conducted the ANOVA tests in order to compare accuracy, sensitivity, specificity, and precision achieved in both scenarios. The tests were performed for each frequency band and classification measure separately. The results are presented in Table 3. As the analysis shows, there are no statistical differences between specificity and precision between both scenarios. However, results obtained for sensitivity were significantly higher for the first scenario for all frequency bands and their combinations, except for the β (for details, see Table 4 and Table 5). The results also indicate that for the first scenario, accuracy is significantly higher for the different sets of bands, except for the single θ, α, β bands, and combination of the β γ bands, where the test did not provide enough evidence for differences.

**Figure 5:**
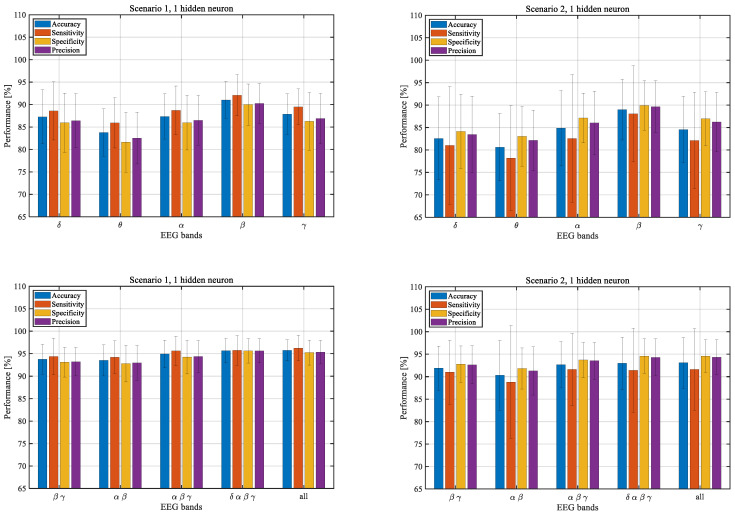
Performance of the system with one hidden neuron for EEG frequency bands for the first (**left**) and the second (**right**) scenario.

In both scenarios, the single frequency band analysis showed the highest accuracy for the β band, followed by α and γ. Differences in sensitivity, specificity, and precision values correspond to changes in accuracy. The values of the classification measures increase when two or more frequency bands are combined. Table 4 and Table 5 show the averaged values and standard deviations for each measure for the first and the second scenarios. To better illustrate the results, the average values of the measures are also presented in Figure 5.

### 3.3. Differences between EEG Frequency Bands

To determine differences between the results for the different frequency bands, we performed a one-way parametric ANOVA (data followed normal distribution according to the Kolmogorov–Smirnov test for all frequency bands, *p* > 0.13) and a post hoc test using Tukey’s honestly significant difference procedure. The results are presented in Table 6 for the first scenario and Table 7 for the second scenario. The outcomes highlighted in red indicate a statistical difference in accuracy for the defined frequency bands and those in green indicate that there is not enough evidence for it.

In the first scenario (training and testing feature set vectors derived from each recording session), the β frequency band provided the highest accuracy and the lowest standard deviation among the single frequency bands (Table 4 and Table 6). The results of tests show that for the β band there is not enough evidence that the results are different from the results for the γ frequency band and the combination of the β γ and α β frequency bands. However, there are statistical differences between the results for the β and the combinations of three or more frequency bands. The results obtained for the δ, α, and γ frequency bands were similar and show that there is not enough evidence for the differences between them. For the θ band, significantly worse results than for other frequency bands were obtained. The performed tests indicate that after combining the β with the α or γ band, a further increase in the number of frequency bands did not provide sufficient evidence that the results are significantly better.

Training and testing feature set vectors were separated by recording sessions during the second scenario (Table 5 and Table 7). Similar to the first scenario, the results for the single frequency bands indicate that the highest accuracy and the lowest standard deviation among single frequency bands were obtained for the β band. The test did not provide enough evidence for differences between the single β frequency band and combinations of two or more bands for the second scenario. The β frequency band significantly differs only from the θ and δ frequency bands. The second-best performance was found for the α and γ frequency bands. Compared to the first scenario, the δ band performed worse. Again, the worst results were obtained for the θ band.

### 3.4. External Impostor Attack

In the last part of our study, we verified the robustness of the verification system to an external impostor attack. For each of the verified subjects (29), we performed the attack using 552 trials (originating from 23 participants, for every 24 attempts of 7.5 s). None of these 23 impostors used to perform the attack attempt had been used to train the networks.

The results of the attacks for the first and second scenarios are presented in Table 8 and Table 9, respectively. Ultimately, 16,008 attack attempts were conducted (552 attacks for each of the 29 dedicated neural networks). The results are presented for all sets of frequency bands (rows) and all analyzed numbers of hidden neurons (columns). Although we did not find sufficient evidence for statistically significant changes in the number of hidden neurons in previous analyses (see Section 3.1), we decided to repeat this investigation for the external impostor attack. We wanted to exclude the possibility that entirely external data would bring any additional critical input.

For both scenarios, we performed the two-way ANOVA to compare the number of false positives for different numbers of hidden neurons and considered EEG frequency bands. There is not enough evidence for differences between the results for the analyzed numbers of hidden neurons in both scenarios (first one: ANOVA F-test (9, 2800) = 0.6, *p* = 0.80, second: F (9, 2800) = 0.74, *p* = 0.67). However, a strong influence of the examined EEG frequency bands on the achieved results was found (first scenario: F (9, 2800) = 114.47, *p* = 0, second: F (9, 2800) = 123.44, *p* = 0). In both scenarios, the performed test shows no significant interactions between the considered factors (first scenario: F (9,2800) = 0.21, *p* = 1, second: F (9, 2800) = 0.27, *p* = 1).

Analyzing the results obtained for one hidden neuron, the best performance was achieved for the β and δ frequency bands, followed by γ and α. The worst performance was obtained for the θ frequency band. The best results were achieved for the combination of all frequency bands. There was not enough evidence that the results for neural networks trained using the two considered scenarios are different (ANOVA F-test (1, 56) < 1.47, *p* > 0.23).

## 4. Discussion

The analysis of the classification performance of the EEG-based verification was conducted in two scenarios. In the first, different parts of each recording session were used to create the training and testing datasets. In the second one, the datasets were separated by recording sessions.

The main findings of our study concern the effect of using spectral features from individual EEG bands on the verification results. Among the single frequency bands, in both scenarios, the best results were obtained for the β frequency band, followed by the bands directly adjacent to the β band, i.e., α and γ (Table 4 and Table 5). Comparable results with the α and γ frequency bands were also obtained for the δ frequency band in the first scenario. It may suggest the impact of technical conditions on the classifications—the δ frequency band is usually affected by sweat and temperature, resulting in increased electrode resistance. Additionally, in the second scenario, the highest average standard deviation was observed in the δ frequency band. In both cases, the θ frequency band had the worst performance.

It should be noted that the best results in both scenarios for single frequency bands were observed for the β band, which is often considered the physiological marker of attention [39,40]. Although classification results of spectral features derived from the α band provided comparable results, it is highly susceptible to various manipulations [41], making it more vulnerable to falsification. Therefore, it seems less practical for application in human verification protocols. Higher EEG frequencies (such as high β or γ) overlap with the muscle activity [42], which makes the protocols based on these frequencies susceptible to manipulations [42,43,44]. Therefore, single frequencies for person verification should be used with caution.

The high and stable classification results observed in our experiment for the β frequency band might be related to the unique role of these oscillations in global neural network synchronization. Strong long-range connections in β and γ have been found to be highly stable [45,46,47], less prone to interferences, and less energetically demanding [45,46,47].

While analyzing greater numbers of frequency bands, the improvement in the verification and a decrease in the differences between both scenarios can be observed. However, it may be caused by the overall improvement of classification measures. In the second scenario, the values of standard deviations are almost twice as large as in the first scenario than in the second one (compare the results in Table 1 and Table 2). With more frequency bands used, the results become more similar to each other, but still, the sensitivity is statistically higher in the first scenario (compare the results in Table 3, Table 4 and Table 5).

The performed studies show that there is not enough evidence for the influence of the number of hidden neurons in the range from 1 to 10 on classification accuracy. It may suggest that the use of one hidden neuron is sufficient for robust verification. This finding was further confirmed by the external impostor attack, where we could not find an advantage of additional hidden neurons.

The conducted studies present the significant differences in classification measures obtained for the two different scenarios of the verification process. The sensitivity of the classification in the first scenario is significantly higher than in the second one. These differences can be found using the single EEG frequency bands (except for the β band). Therefore, special attention must be paid to the results focused on interpersonal differences, which do not seem to represent practical application problems.

Although some findings on the verification systems in the current literature seem to outperform ours, the data and applied testing scenarios used for their assessment are often very different from ours. The exorbitant results obtained in the studies may result from the fact that the test signals closely resemble training signals. For example, we found studies where testing and training data came from the same one-minute segments. By reviewing results reported in other papers [22,23], one can notice that as the number of examinations used in the analysis increases, the results deteriorate. For instance, in [24], using simple cross-correlation values of PSD features of the gamma frequency band managed to achieve an equal error rate (EER) of 0.0196 among 109 subjects using eyes-closed and eyes-open resting-state EEG recordings. However, in this experiment, the authors considered one-minute EEG signals from individual participants, divided into eight segments. Out of these eight segments, seven were used for training and one for testing. For the same public database, other authors [10] achieved an EER of 0.016. The authors divided one minute of EEG signals into eight segments and discarded the first one, yielding six segments used for training and one for testing. In both studies, the authors used the same database, where there was only one EEG recording for each person (one minute with eyes open and one minute with eyes closed). Signals used for training and testing were treated as different observations originating from the same one-minute source. The work continued later [36] using a correlation-based classifier and a frequency-weighted power (FWP), for the same database, the authors obtained an EER of 0.0039 from eyes-closed resting-state EEG using 20 electrodes. The considered problem of using data from an insufficient number of sessions is still of interest, as evidenced by recently published articles on EEG in biometrics [13,15,28,29,30,33,48,49]. Since the considered problem is also related to other paradigms, the resting-state data may serve as a reference for other paradigms. Examining the EEG-based person verification efficiency in repeatedly time-correlated studies performed on different days is extremely rare and provides new insight. Our database provided 603 examinations (29 participants each with 20 sessions performed on different days and 23 additional impostors with one session).

Coming back to our results, there was not enough evidence for significant differences in specificity between the two scenarios for the external impostor attack discussed in our paper (Table 8 and Table 9). Although we did not find enough evidence for differences in the specificity of the analyzed solutions between scenarios (Table 3), we found significant differences in the sensitivity. It may suggest a large diurnal variability of the EEG signals due to the psychophysical state of the participant. The effect of the technical aspects of measurements is also possible, for instance, due to differences in the resistance and placement of electrodes or environmental cleanliness. Such variability seems natural under the operating conditions of the real-life system (represented by the second scenario in our investigations) and should be considered when evaluating the accuracy and reliability of tested solutions. Therefore, EEG recordings should be collected over a more extended period of time. Such an approach would provide independence of biometric recognition results from various daily psychophysical states or technical conditions. We believe that the longer the data collecting period is, the more reliable the system would be. The first scenario should be considered only as a reference since, in practical applications, the biometric recognition of individuals should be possible using the already-trained networks without frequent cyclical enrollment. However, their update should be carried out from time to time.

## 5. Conclusions

The results presented in this paper confirm the applicability of people verification using spectral features of individual EEG frequency bands as features of shallow neural networks. The verification systems using short segments of EEG signal in the resting state can be employed in a variety of areas, ranging from medical applications for patient identity control to sophisticated military applications, e.g., for controlling access to secret information, as a component of a multi-stage verification process, or continuous control for various remotely controlled installations, devices, or vehicles.

It should be stressed that the presented results were obtained using a large number of EEG sessions (603) spread over approximately two months, which resembles practical applications and makes the presented results unique. Another important aspect of our study is the comparison of two different scenarios of training and testing the networks. The second scenario seems to better reflect the practical application and should be considered as the primary method for evaluating verification protocols. In further research, we are going to study how the number of sessions (acquired on separate days) taken during the training influence the results of the EEG-based person verification.

Future research plans are related to the analysis of the influence of signals registered by individual EEG electrodes on the multiple-session verification efficiency to simplify the EEG signal acquisition device and both the training process and the verification procedure itself. Minimizing the number of electrodes needed to perform the biometric recognition would enable the use of existing commercial headsets to acquire EEG signals or to develop a dedicated or more convenient one instead of using medical-grade helmets which are time-consuming (as described in [16]). Another direction of our investigation concerns the length of EEG signal fragments needed to be analyzed in the biometric recognition system in order to obtain the excepted efficiency.

## Figures and Tables

**Table 1:** Mean and standard deviation of accuracy for selected EEG frequency bands (fb) and a different number of hidden neurons (hn). Results for the first scenario in which feature set vectors for the training and testing datasets were derived from each recording session.

	hn	1	2	3	4	5	6	7	8	9	10
fb											
δ		87.3 ±6.0	87.4 ±5.8	87.5 ±5.3	88.2 ±5.6	87.9 ±5.3	89.0 ±5.1	88.9 ±5.1	88.3 ±5.4	88.8 ±5.1	88.9 ±5.3
θ		83.8 ±5.3	83.4 ±5.4	84.3 ±4.7	83.8 ±4.4	84.1 ±4.9	84.3 ±4.8	84.1 ±5.1	84.0 ±5.0	84.2 ±5.2	83.8 ±5.3
α		87.3 ±5.0	87.6 ±5.1	87.5 ±4.9	88.0 ±5.0	88.0 ±4.9	87.8 ±5.0	87.5 ±4.5	87.6 ±4.8	87.6 ±4.7	87.3 ±5.4
β		91.0 ±4.1	91.5 ±4.1	92.1 ±3.9	92.0 ±3.8	91.9 ±3.9	91.9 ±4.1	92.1 ±3.5	91.9 ±3.8	92.4 ±3.9	92.2 ±3.8
γ		87.9 ±4.5	88.1 ±5.0	88.1 ±5.0	87.9 ±4.9	87.5 ±5.0	88.0 ±4.5	87.5 ±4.7	87.3 ±5.3	87.6 ±5.0	87.0 ±5.3
β γ		93.7 ±3.3	93.2 ±3.7	93.3 ±3.3	93.6 ±3.3	93.5 ±3.5	93.8 ±3.1	94.0 ±2.8	94.1 ±3.0	93.7 ±3.1	93.7 ±3.1
α β		93.5 ±3.5	93.5 ±3.5	93.6 ±3.7	93.6 ±3.4	93.6 ±3.6	93.4 ±4.0	93.7 ±3.6	94.0 ±3.1	94.1 ±3.3	93.8 ±3.4
α β γ		94.9 ±3.1	94.7 ±2.8	94.9 ±3.1	94.8 ±3.0	94.6 ±3.0	94.9 ±2.9	94.8 ±3.3	94.3 ±2.9	94.4 ±3.0	94.6 ±2.6
δ α β γ		95.7 ±2.7	95.4 ±2.7	95.3 ±2.6	95.4 ±2.6	95.0 ±2.9	95.4 ±2.8	95.3 ±2.8	95.2 ±2.5	95.3 ±2.5	95.3 ±2.8
All		95.7 ±2.3	95.5 ±3.0	95.6 ±2.7	95.4 ±2.9	95.6 ±2.8	95.6 ±2.8	95.4 ±2.9	95.2 ±2.9	95.2 ±2.9	95.5 ±2.8

**Table 2:** Mean and standard deviation of accuracy for selected EEG frequency bands (fb) and a different number of hidden neurons (hn). Results for the second scenario in which feature set vectors for the training and testing datasets were derived from separate recording sessions.

	hn	1	2	3	4	5	6	7	8	9	10
fb											
δ		82.6 ±9.2	82.9 ±8.6	82.7 ±8.6	82.0 ±8.6	82.7 ±9.4	83.6 ±8.5	82.7 ±9.1	82.9 ±8.7	83.3 ±8.7	82.8 ±8.7
θ		80.6 ±7.5	80.7 ±7.2	81.3 ±7.3	80.6 ±7.6	80.6 ±7.4	80.6 ±7.8	80.7 ±7.5	80.8 ±6.9	80.9 ±6.8	80.9 ±7.3
α		84.8 ±8.4	85.0 ±7.5	85.2 ±8.4	85.1 ±7.6	85.3 ±7.5	85.8 ±7.0	85.5 ±7.0	86.0 ±8.1	85.8 ±7.2	85.8 ±7.3
β		89.0 ±6.8	89.4 ±5.9	89.0 ±6.8	89.5 ±6.6	89.5 ±6.0	89.7 ±6.3	89.8 ±5.8	89.7 ±6.3	90.0 ±6.0	90.0 ±5.3
γ		84.6 ±7.4	84.9 ±7.4	84.7 ±7.3	83.8 ±7.7	84.1 ±7.3	84.6 ±7.2	84.3 ±7.3	83.9 ±7.3	83.3 ±7.1	84.2 ±6.9
β γ		91.9 ±4.9	91.0 ±5.8	91.4 ±5.4	91.2 ±5.5	91.4 ±5.0	91.4 ±5.5	91.5 ±5.2	91.8 ±5.2	91.4 ±5.6	91.2 ±5.7
α β		90.3 ±7.7	90.7 ±7.0	90.8 ±7.1	91.0 ±6.6	91.4 ±6.5	91.5 ±5.7	91.7 ±6.2	91.4 ±6.3	91.4 ±6.3	91.6 ±6.7
α β γ		92.7 ±5.1	92.4 ±5.4	92.4 ±6.1	93.0 ±5.5	92.6 ±5.7	92.2 ±6.1	92.1 ±6.4	92.6 ±6.0	92.9 ±4.5	92.0 ±6.0
δ α β γ		93.0 ±5.8	93.0 ±5.8	93.3 ±5.8	93.1 ±5.4	92.9 ±5.1	93.0 ±5.6	93.0 ±5.6	92.8 ±5.8	93.0 ±5.9	93.0 ±5.1
All		93.1 ±5.6	93.1 ±5.8	93.4 ±5.2	93.3 ±5.6	92.9 ±5.8	93.0 ±6.1	92.6 ±5.8	93.2 ±5.4	92.9 ±6.0	92.9 ±5.2

**Table 3:** *p*-values F (1, 56) obtained using series of ANOVA between two scenarios. Results marked in green mean that there is not enough evidence for differences between scenarios, in red, there is. The results for the one hidden neuron.

EEG Bands	ACC (%)	SEN (%)	SPEC (%)	PREC (%)
δ	0.03	7.0 × 10^−3^	0.36	0.13
θ	0.07	2.1 × 10^−3^	0.41	0.81
α	0.17	0.03	0.46	0.80
β	0.17	0.07	0.95	0.65
γ	0.04	1.1 × 10^−3^	0.66	0.67
β γ	0.10	0.03	0.74	0.56
α β	0.05	0.03	0.39	0.18
α β γ	0.05	0.02	0.61	0.41
δ α β γ	0.03	0.02	0.23	0.15
All	0.02	0.01	0.44	0.26

**Table 4:** Classification measures obtained for the first scenario, in which training and testing feature set vectors originated from each session.

EEG Bands	ACC (%)	SEN (%)	SPEC (%)	PREC (%)
δ	87.3 ± 6.0	88.6 ± 6.5	85.9 ± 6.6	86.4 ± 6.0
θ	83.8 ± 5.3	85.9 ± 5.6	81.6 ± 6.6	82.5 ± 5.8
α	87.3 ± 5.0	88.7 ± 5.4	86.0 ± 6.1	86.5 ± 5.5
β	91.0 ± 4.1	92.1 ± 4.6	90.0 ± 4.6	90.3 ± 4.4
γ	87.9 ± 4.5	89.5 ± 4.0	86.3 ± 6.4	86.9 ± 5.6
β γ	93.7 ± 3.3	94.4 ± 4.1	93.1 ± 3.3	93.2 ± 3.2
α β	93.5 ± 3.5	94.2 ± 3.6	92.8 ± 4.1	92.9 ± 3.9
α β γ	94.9 ± 3.1	95.6 ± 3.2	94.2 ± 3.7	94.4 ± 3.6
δ α β γ	95.7 ± 2.7	95.7 ± 3.3	95.6 ± 2.7	95.6 ± 2.7
All	95.7 ± 2.3	96.2 ± 2.9	95.2 ± 2.8	95.3 ± 2.6

**Table 5:** Classification measures obtained for the second scenario, in which training and testing feature set vectors are from different sessions.

EEG Bands	ACC (%)	SEN (%)	SPEC (%)	PREC (%)
δ	82.6 ± 9.2	81.0 ± 13.1	84.1 ± 8.3	83.5 ± 8.5
θ	80.6 ± 7.5	78.2 ± 11.7	83.0 ± 6.6	82.1 ± 6.7
α	84.8 ± 8.4	82.6 ± 14.2	87.1 ± 5.6	86.1 ± 7.1
β	89.0 ± 6.8	88.0 ± 10.7	89.9 ± 5.5	89.7 ± 5.8
γ	84.6 ± 7.4	82.1 ± 10.7	87.0 ± 6.0	86.2 ± 6.6
β γ	91.9 ± 4.9	91.0 ± 7.1	92.8 ± 4.0	92.6 ± 4.2
α β	90.3 ± 7.7	88.8 ± 12.5	91.8 ± 4.6	91.3 ± 5.3
α β γ	92.7 ± 5.1	91.6 ± 8.0	93.7 ± 3.9	93.5 ± 4.1
δ α β γ	93.0 ± 5.8	91.4 ± 9.3	94.5 ± 3.8	94.3 ± 4.1
All	93.1 ± 5.6	91.6 ± 9.0	94.6 ± 3.6	94.3 ± 3.9

**Table 6:** *p*-values F (9, 280) obtained using pair-wise Tukey’s honestly significant difference procedure between different frequency bands. The results for the first scenario when training and testing feature set vectors were from the same recording. The red indicate a statistical difference in accuracy, the green, that there is no enough evidence for it.

EEG Bands	δ	θ	A	β	γ	β γ	α β	α β γ	δ α β γ	All
δ		0.04	1.00	0.02	1.00	2.5 × 10^−7^	6.1 × 10^−7^	1.3 × 10^−7^	1.3 × 10^−7^	1.3 × 10^−7^
θ			0.03	1.3 × 10^−7^	0.01	1.3 × 10^−7^	1.3 × 10^−7^	1.3 × 10^−7^	1.3 × 10^−7^	1.3 × 10^−7^
α				0.02	1.00	3.1 × 10^−7^	8.4 × 10^−7^	1.3 × 10^−7^	1.3 × 10^−7^	1.3 × 10^−7^
β					0.10	0.28	0.42	0.01	9.0 × 10^−4^	6.7 × 10^−4^
γ						3.4 × 10^−6^	1.1 × 10^−5^	1.3 × 10^−7^	1.3 × 10^−7^	1.3 × 10^−7^
β γ							1.00	0.99	0.76	0.71
α β								0.95	0.60	0.56
α β γ									1.00	1.00
δ α β γ										1.00
All										

**Table 7:** *p*-values F (9, 280) obtained using pair-wise Tukey’s honestly significant difference procedure between different frequency bands. The results for the second scenario when training and testing feature set vectors were separated by recording sessions. The red indicate a statistical difference in accuracy, the green, that there is no enough evidence for it.

EEG Bands	δ	θ	α	β	γ	β γ	α β	α β γ	δ α β γ	All
δ		0.99	0.97	0.02	0.99	1.7 × 10^−5^	1.0 × 10^−3^	1.8 × 10^−6^	7.4 × 10^−7^	5.5 × 10^−7^
θ			0.39	2.2 × 10^−4^	0.49	1.6 × 10^−7^	5.6 × 10^−6^	1.3 × 10^−7^	1.3 × 10^−7^	1.3 × 10^−7^
α				0.42	1.00	4.8 × 10^−3^	0.08	8.5 × 10^−4^	3.9 × 10^−4^	2.9 × 10^−4^
β					0.32	0.86	1.00	0.59	0.47	0.43
γ						2.6 × 10^−3^	0.05	4.2 × 10^−4^	1.9 × 10^−4^	1.4 × 10^−4^
β γ							1.00	1.00	1.00	1.00
α β								0.96	0.91	0.89
α β γ									1.00	1.00
δ α β γ										1.00
All										

**Table 8:** The percentage of successful impostor attacks for the first scenario.

	hn	1	2	3	4	5	6	7	8	9	10
fb											
δ		14.96	15.18	14.51	15.24	16.66	15.26	14.99	14.74	14.86	15.27
θ		22.08	21.08	21.18	22.16	21.90	22.29	20.72	22.79	22.50	22.74
α		17.83	17.95	18.04	16.82	18.07	17.49	17.45	18.95	18.87	19.18
β		14.06	13.59	13.24	13.13	13.27	14.38	13.64	13.29	13.32	13.85
γ		18.06	16.60	17.63	17.84	17.57	17.22	17.99	18.56	17.66	19.31
β γ		11.94	12.45	12.08	11.63	12.18	11.76	13.12	11.31	12.66	12.27
α β		11.83	12.41	11.77	11.38	12.18	12.34	12.77	12.52	12.20	11.41
α β γ		10.31	10.59	10.44	11.03	11.34	11.03	10.40	11.44	11.11	11.42
δ α β γ		9.00	9.16	9.69	10.19	9.82	8.85	9.41	10.57	9.80	10.63
All		8.41	8.48	9.09	9.76	8.88	9.05	9.41	9.41	10.59	10.43

**Table 9:** The percentage of successful impostor attacks for the second scenario.

	hn	1	2	3	4	5	6	7	8	9	10
fb											
δ		14.99	15.39	16.02	15.83	16.05	15.11	15.31	15.95	16.24	15.74
θ		21.82	22.22	21.35	21.61	22.75	20.62	21.21	20.96	21.85	21.55
α		16.32	16.35	16.62	18.51	17.36	16.95	17.70	18.40	19.28	18.36
β		14.61	13.62	12.94	13.87	13.52	14.07	13.54	14.22	14.27	13.61
γ		15.87	16.49	16.87	16.76	17.49	18.59	17.60	17.56	17.60	18.82
β γ		11.98	12.55	12.52	11.79	11.24	12.28	11.88	12.39	12.64	11.78
α β		10.96	11.22	11.88	11.29	11.65	12.52	11.67	11.73	11.36	11.52
α β γ		10.48	10.14	10.11	9.83	11.13	10.97	11.36	11.69	10.56	10.93
δ α β γ		9.24	9.35	8.56	8.50	9.65	9.79	9.12	8.95	9.57	10.41
All		8.46	8.24	8.32	9.25	8.52	9.39	9.25	9.11	10.10	10.48

**Figure A1:**
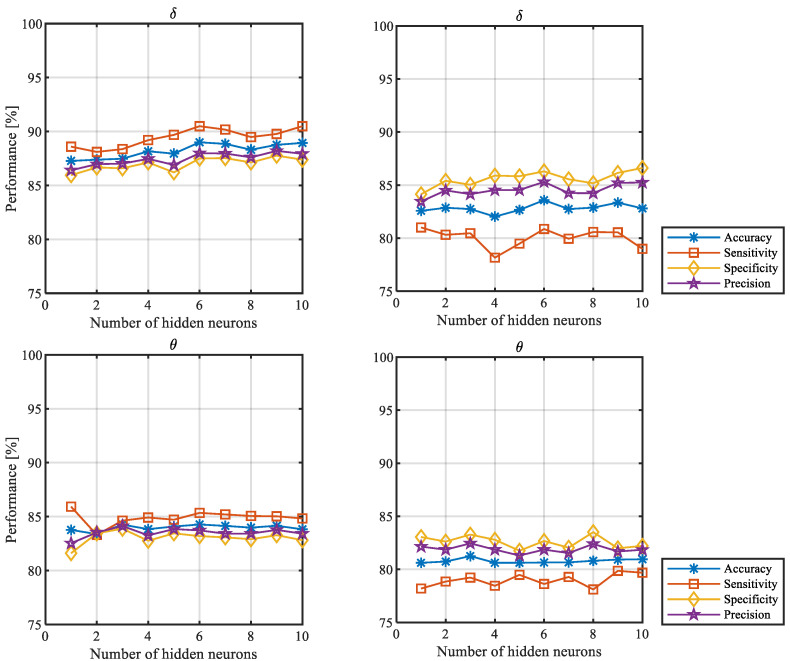
Network classification measures for selected single frequency bands (δ, θ), where the training and testing feature set vectors were separated by different parts of the same sessions (**left**, the first scenario) and different sessions (**right**, the second scenario).

**Figure A2:**
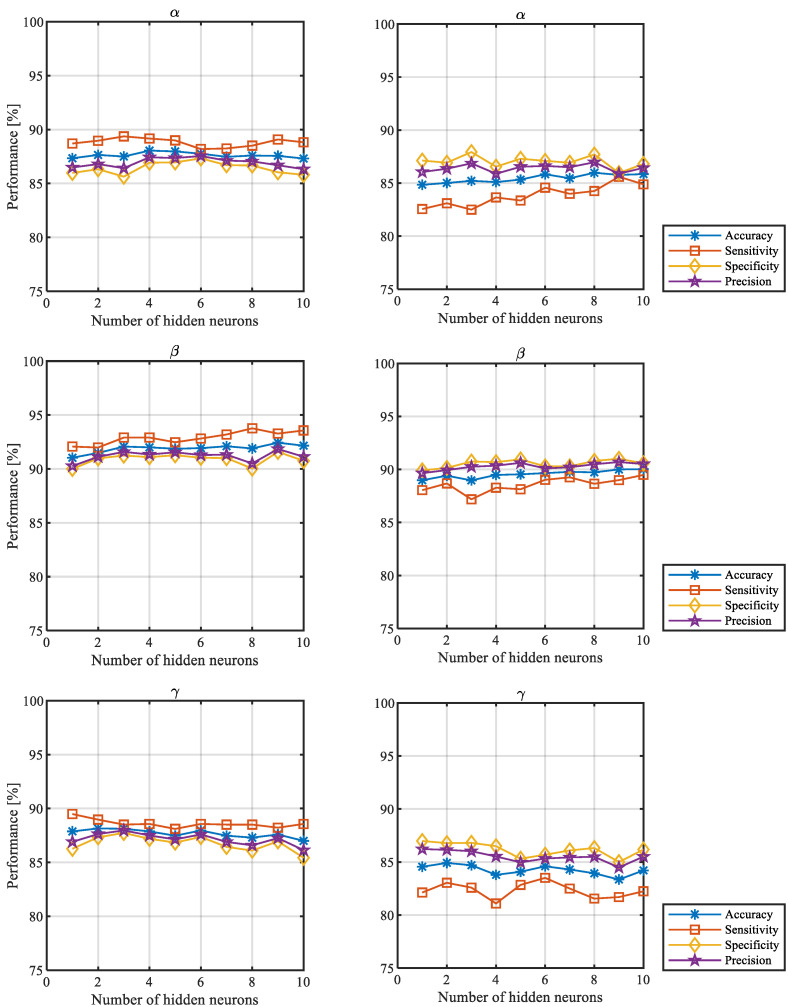
Network classification measures for selected single frequency bands (α, β, γ), where the training and testing feature set vectors were separated by different parts of the same sessions (**left**, the first scenario) and different sessions (**right**, the second scenario).

**Figure A3:**
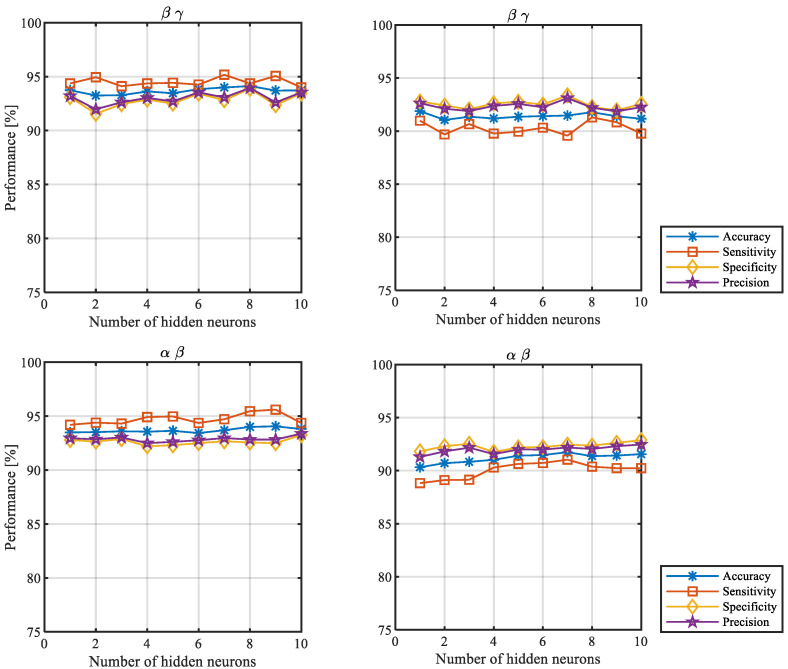
Network classification measures for selected combinations of frequency bands, where the training and testing feature set vectors were separated by different parts of the same sessions (**left**, the first scenario) and different sessions (**right**, the second scenario).

**Figure A4:**
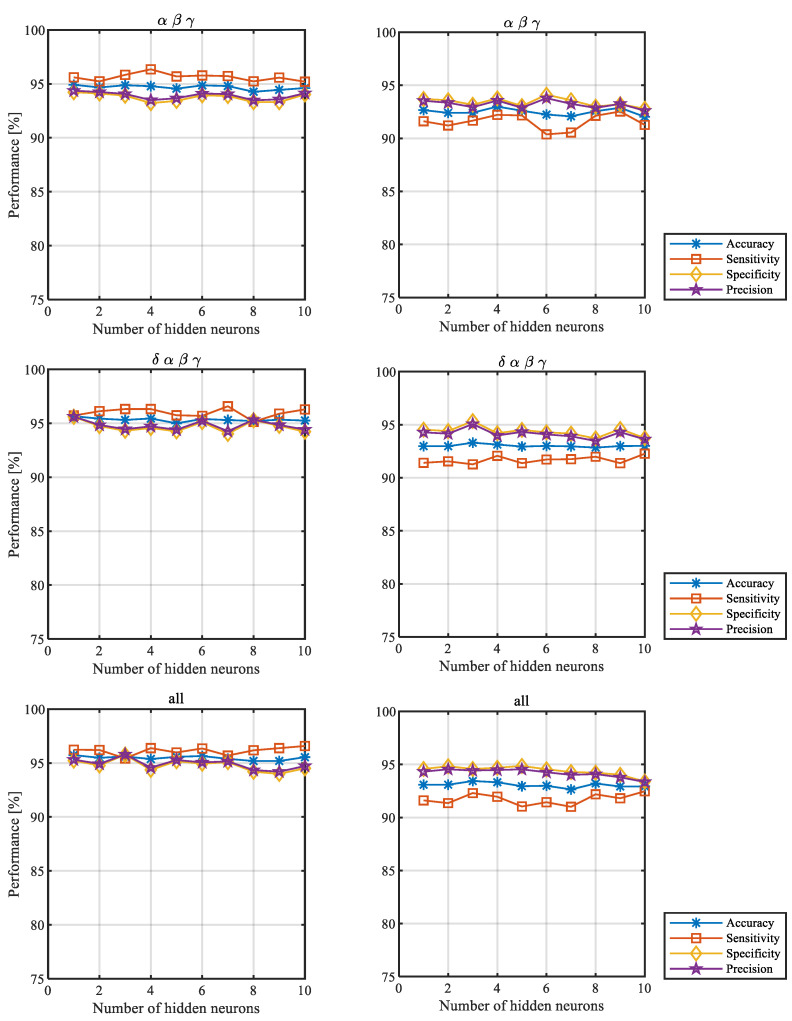
Network classification measures for selected combinations of many frequency bands, where the training and testing feature set vectors were separated by different parts of the same sessions (**left**, the first scenario) and different sessions (**right**, the second scenario).

## Data Availability

Data available on request.

## Appendix A

The figures below show the classification measures of the verification depending on the number of hidden neurons. We traced the results for the following performance measures: accuracy, sensitivity, specificity, and precision, for all EEG frequency bands and their combination considered in Section 3.
